# Providing open access data online to advance malaria research and control

**DOI:** 10.1186/1475-2875-12-161

**Published:** 2013-05-16

**Authors:** Catherine L Moyes, William H Temperley, Andrew J Henry, Clara R Burgert, Simon I Hay

**Affiliations:** 1 Department of Zoology, Spatial Ecology and Epidemiology Group, Tinbergen Building, South Parks Road, Oxford, OX 1 3PS, UK; 2 Joint Research Centre of the European Commission, Ispra VA JRC 21027, Italy; 3 MEASURE DHS Project, ICF International, 11785 Beltsville Dr, Suite 300, Calverton, MD 20705, USA

**Keywords:** Parasite rate, Vector occurrence, Duffy, G6PD, Sickle haemoglobin, Plasmodium falciparum

## Abstract

**Background:**

To advance research on malaria, the outputs from existing studies and the data that fed into them need to be made freely available. This will ensure new studies can build on the work that has gone before. These data and results also need to be made available to groups who are developing public health policies based on up-to-date evidence. The Malaria Atlas Project (MAP) has collated and geopositioned over 50,000 parasite prevalence and vector occurrence survey records contributed by over 3,000 sources including research groups, government agencies and non-governmental organizations worldwide. This paper describes the results of a project set up to release data gathered, used and generated by MAP.

**Methods:**

Requests for permission to release data online were sent to 236 groups who had contributed unpublished prevalence (parasite rate) surveys. An online explorer tool was developed so that users can visualize the spatial distribution of the vector and parasite survey data before downloading it. In addition, a consultation group was convened to provide advice on the mode and format of release for data generated by MAP’s modelling work. New software was developed to produce a suite of publication-quality map images for download from the internet for use in external publications.

**Conclusion:**

More than 40,000 survey records can now be visualized on a set of dynamic maps and downloaded from the MAP website on a free and unrestricted basis. As new data are added and new permissions to release existing data come in, the volume of data available for download will increase. The modelled data output from MAP’s own analyses are also available online in a range of formats, including image files and GIS surface data, for use in advocacy, education, further research and to help parameterize or validate other mathematical models.

## Background

The Malaria Atlas Project (MAP) was established in 2005 to provide evidence-based estimates of populations at malaria risk using a cartographic approach [1]. Then and now, parasite prevalence surveys that measure the proportion of a community with parasites in their blood (commonly referred to as PR or parasite rate surveys) provide the bulk of the global information available on malaria endemicity. In the intervening years, the MAP group conducted extensive searches of the published literature including peer reviewed journal articles and published reports, and contacted hundreds of malaria control programmes, research groups, Ministries of Health and aid agencies to request access to their survey data. All survey results received were disaggregated to individual sites, individual dates and individual parasite species; duplicates were excluded and a precise geoposition was calculated for each site (where it was not provided with the data). In 2010, the resulting database contained clean and geopositioned *Plasmodium falciparum* prevalence survey records for 22,249 unique site-date combinations. An interim dataset was used to produce MAP’s 2007 global map of *P. falciparum* endemicity [2] and the full dataset was used for the 2010 version, which incorporated vastly improved methods, as well as the addition of covariates [3]. These data have also been used to undertake national analyses tailored to individual country needs, for example for Somalia [4], Kenya [5], Indonesia [6,7], Djibouti [8] and Sudan [9,10]. Since 2010, new data have been collated and geopositioned and the current total is 24,210 survey records. Of these, 9,970 were used in MAP’s modelling work to estimate the global endemicity of *Plasmodium vivax* malaria [11,12].

From the outset, MAP undertook to ensure all data output by MAP’s models would be made available in the public domain [13] and details of the scripts used to run MAP’s models were made available on GitHub under a GNU Public Licence for open source code [14]. A large portion (80%) of the parasite survey data collated was unpublished, and none of these data had been generated by MAP, so a new exercise was initiated to seek data release permissions from the original data sources [15].

In addition, MAP launched a parallel exercise to collate anopheline mosquito surveys that reported the occurrence and/or absence of the dominant *P. falciparum* malaria vector species [16]. These data were almost exclusively collated from published, peer-reviewed journal articles. Again survey results were disaggregated to individual sites, individual dates and individual species (or species complex/subgroup); duplicates were excluded and a precise geoposition was calculated for each site (where it was not provided with the data). This exercise yielded survey results for 30,324 site-date-species combinations extracted from 2,060 published articles. These data were used to estimate the spatial distribution of 41 dominant vector species in three distinct regions of the world [17-20].

A suite of work on human genetic variants of relevance to malaria endemic countries is also underway and MAP has committed to making the survey data collated for this work available online [21-23]. This release is described fully in a separate publication [24] and is summarized here.

This article describes both the survey data and the modelled data that can now be found online and outlines the mechanisms of release that were developed with the aim of making the datasets accessible and readily located, intelligible, assessable and useable [25].

## Methods

### The database

The parasite prevalence and vector occurrence survey datasets used by MAP have been described in full in [3] and [17-19], respectively, and these papers detail how the data were identified. The survey data are held in three sections of a relational database (built using the PostgreSQL management system). One section contains tables that describe the site; providing a latitude, longitude (in decimal degrees to five decimal places) for each point location. Data quality checks were performed to ensure that the coordinates fell within country boundaries and on land as defined by MAP’s master grid using a WGS84 coordinate reference system. The sites were then checked to identify pairs of sites within 1,000 m of one another. Of these, genuine duplicates were assigned the same site identifier. The final check looked for spatio-temporal duplicates. If the same, or an overlapping community, was surveyed within three months then only the first survey record was retained. A second section of the database contains a set of bibliographic tables and each bibliographic source is classified as either published, permission to release or confidential.

**Figure 1:**
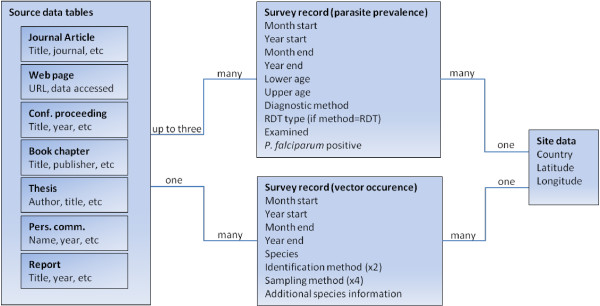
**Simplified schematic of the MAP database.** The boxes on the left represent the tables that make up the bibliographic dataset. They are linked to the survey tables represented by the central boxes in Figure 1 and these are in turn linked to the site tables represented by the box on the right of the Figure. A full list of fields within the survey and site tables that are available for download is given. A subset of example fields from the bibliographic tables is given within the boxes on the left.

The third section of the database contains the survey data itself and the fields included for parasite prevalence and vector occurrence surveys are shown in Figure 1. Within the context of this database, a single survey result at a specific site and date is one data record. For example, 14 *P. falciparum* positives confirmed by microscopy out of 120 community members aged 1–10 years old in Goma village examined from 1 to 30 June 2008 would constitute a single record. Only complete survey data were included in the database, with one rarely applied exception. Parasite prevalence surveys that provided a single figure for prevalence rather than the numerator (positives) and denominator (number examined), were assumed to have a number examined of 30 for the purposes of MAP’s modelling work [3]. Dates were inferred from seasons where necessary and if a date was missing altogether the survey was not included.

The links between these database sections are shown in the simplified schema in Figure 1. A source may link to many survey records and a survey record may contain data derived from more than one source. For example, the prevalence results may be given in a paper (source 1) while the age of the subjects was provided separately by the authors in a personal communication (source 2). The confidentiality level of a survey record is defined by the highest confidentiality level among the sources it is linked to, for example if a survey record is linked to a journal article classified as ‘published’ and a personal communication classified as ‘confidential’ then the survey record is treated as ‘confidential’.

### Obtaining permission to release the data

The vast majority of vector occurrence data were collated from published sources and the remaining data came with permission to share. In contrast, of the 24,210 clean parasite prevalence survey records held in the MAP database in 2012, only 3,885 records had previously been published. A total of 540 unpublished datasets contributed by 236 groups were identified and permission was requested from each group to release the data freely online. This also provided an opportunity to check the source details (i.e. the citation) with the data contributors. The only commitments given to the data contributors were that unpublished data would not be released until permission was received and that the citation(s) for the original data source would be provided with the released data.

### Development of an online data explorer

The Data Explorer was designed to allow users with low bandwidths to visualize the geographical spread of the survey data overlain on a Google Maps base map and to download these data. Additional functionality was included to allow users to i) view/download parasite prevalence survey data by country and vector occurrence data by species, ii) view locations of all surveys or surveys available for download only, iii) to provide a dynamic summary of the number of surveys displayed on the map as the view changes, and iv) to display MAP’s estimates of parasite prevalence underneath the survey locations. The Data Explorer was developed using Google Web Toolkit, which allowed for the development of client-side web applications in pure Java with no Flash. The Data Explorer was tested with Google Chrome, Mozilla Firefox, Internet Explorer (including IE6), Safari and Opera. The Data Explorer was not developed for use on mobile devices.

### Consultation on releasing model outputs

Targeted questions were sent to 26 stakeholders including malaria control programme managers, global health policy advisors, malaria researchers, GIS users and malaria modellers asking what data they would find useful for their own work and in which formats. The answers were collated and where possible all preferences were accommodated or the majority view was used.

### Production of publication-quality maps

In order to provide online users with several thousand map images of publication quality, a bespoke software program was developed in Java. The software allows batch production of map images with good quality type-setting allowing dynamic legends, production of high resolution PDFs, easy overlaying of transparent images and the ability to reconfigure grids/scale bars for multiple resolutions. This enabled us to produce global, regional and national map variants from each set of modelled data.

### Terms of access and tracking use

All of the parasite prevalence survey records used in MAP’s work are aggregate data in the sense that a single record covers multiple individuals (e.g. 8 out of 60 community members were infected). MAP does not hold any individual subject data, the data held does not include sensitive information about a community (in the way an equivalent community HIV/AIDS rate might be viewed as sensitive or a survey of the prevalence of genes linked to alcoholism) and the vector data does not include any human data at all. There were, therefore, no ethical imperatives to protect the data by restricting access.

No systems were developed that would impede access, such as applications for authorization, authentication, registration or permissions. The only restriction placed on use of the model outputs is a requirement to cite the source (as detailed in the Creative Commons 3.0 Attribution Unported licence [26]). The survey data are made available on an unrestricted basis. The original source information is provided with each survey record but users are not required to cite an additional MAP publication. The decision to prioritize ease of data access limited the ability to track future data use. Visits to the website are, however, analysed using Google Analytics and publication of new research that has used these online resources will be tracked though citations.

## Results and discussion

### Data and code released

The online Data Explorer contains two separate sets of survey data; vector occurrence data and parasite prevalence data. They are provided separately via two different views (termed “perspectives”) within the Explorer.

All 30,324 vector occurrence survey records, each with a unique site-date-species combination, from 141 countries and representing 41 species [see Additional file 1] can be downloaded via the Data Explorer [27]. Users can view the spatial distribution of the data for the dominant vector species or select a single species to view. The map displaying the survey sites can be zoomed in/out and moved around to show the view desired. The default setting displays both species presence and absence points but the user can select which subset of the data they wish to view. The ‘Display summary’ details the number of sites and unique site-date combinations currently displayed on the map. For further data exploration, users can download the dataset for the species of interest in Microsoft Excel Worksheet format and import it into a package of their choice. Each survey record downloaded is provided with site data. Composite source data taken from the bibliographic database tables are used to provide a journal-style citation.

In addition, qualitative information about the bionomics (behaviour, habitats, preferences) of each of the 41 dominant vector species was placed on the website together with photographs where available [28].

*Plasmodium falciparum* parasite prevalence (PR) survey records have also been placed online via MAP’s Data Explorer [29]. Of the 24,210 survey records with unique site-date combinations, 11,766 records from 76 *P. falciparum* endemic countries are available for download covering 1,420,800 subjects examined [see Additional file 2]. The dataset available for download includes 3,885 survey records that were previously published and a further 7,881 survey records from unpublished sources for which release permission was received. Users can view the geographical spread of 23,095 out of the 24,210 survey records and details of the sources for all 24,210 records are given in the download. A total of 215 survey results have sensitive locations in Myanmar and the locations of these surveys are not displayed by the Data Explorer. The default Data Explorer setting displays the location of both points that are available for download and those that are not, but the user can select which datasets they wish to view and whether they wish to overlay the points onto MAP’s modelled prevalence data. Users can also select a country to view and the ‘Display summary’ provides the number of sites and unique site-date combinations currently shown on the map. Users can download the data itself in Microsoft Excel Worksheet format. Survey records that have not been published and do not have a release permission are not provided, however, the source and site details for these surveys are given in the download unless the site data itself are sensitive.

The current parasite prevalence dataset available for download includes 1,049 survey records provided by the USAID-funded MEASURE Demographic and Health Surveys (MEASURE DHS) repository. These particular parasite datasets can be obtained at the individual subject level from MEASURE DHS directly [30] and at the site level (defined by MEASURE DHS as a cluster) from the MAP Data Explorer. The Data Explorer download includes a MEASURE DHS identifier which allows users to link the aggregate prevalence data provided by MAP to the geographic coordinates of the survey locations that can be obtained from the MEASURE DHS website [30].

The consultation group advised on the information and formats that they would find most useful from the outputs of MAP’s own research and these were placed on a new website [31]. The matrix in Table 1 shows which map images are available in global, regional and national versions for download as both high-resolution PDF files and lower resolution PNG files. Table 1 also shows the population estimates that are available on the site including estimates of populations at *P. falciparum* malaria risk and Duffy negative newborns. The maps currently available include: *P. falciparum* endemicity, reproductive number under control and entomological inoculation rate; Duffy negativity prevalence; the limits of *P. falciparum* and *P. vivax* malaria; and predicted occurrence of 41 dominant vector species. The primary utility of the mapped images is in advocacy. Most respondents wanted to be able to download images to insert into presentations and reports. The mapped images provided were chosen to i) use a consistent format for each region/country enabling comparisons and ii) link to a named peer-reviewed publication to provide credibility. In addition, the map production system was used to produce a suite of bespoke maps based on the same model outputs for the Atlas of Malaria Eliminating Countries, 2011 [32] and a second suite based on World Health Organization (WHO), national and MAP data for the WHO World Malaria Report 2012 [33].

**Table 1:** Products derived from MAP model outputs that are available for download from the MAP website

	**Scale**		
**MAP output**	**Global**	**Regional**	**National**
***P. falciparum*limits**	map	maps	maps
***P. falciparum*endemicity**	map	maps	maps
***P. falciparum*entomological innoculation rate**	map	maps	maps
***P. falciparum*reproductive number under control**	map	maps	maps
**Population at*P. falciparum*risk**			tables of estimates
***P. vivax*limits**	map	maps	maps
**Predicted occurrence of 41 dominant vector species**	full species range maps		maps
**Bionomics for 41 individual dominant vector species**	descriptive text		
**Duffy negativity prevalence (used to estimate*P. vivax*risk)**	map	maps	maps and tables of estimates
***P vivax*endemicity**	map	maps	maps
**Sickle haemoglobin allele frequency**	map	maps	tables of estimates maps
**G6PD deficiency allele frequency**	map	maps	tables of estimates maps
**Multiple vectors**	map	maps	n/a

For the most popular resources, currently *P. falciparum* endemicity, a raster file of the summary model data used to create the online maps (in this instance the mean estimate at each pixel, or 30 arc-seconds/approximately 1 km^2^) is available in both Binary Float and GeoTIFF formats.

Finally, the source code developed to build the online Data Explorer is available on GitHub [34] so that others can use, or conduct further work on, this spatial data dissemination tool.

### Accessing the resource

The main portal for accessing the data types described above is the MAP website where the survey data can be found using the Data Explorer [27,29] and the research results can be found using the Resource Browser [35], by viewing the country profiles [36], by using the site’s own search function or by using public search engines such as Google. Once a user has found the data they want, there are no barriers to access; the site requires no registration, authentication or authorization.

The entire site, including the Data Explorer and Resource Browser, has been designed to be accessible from low bandwidth environments and in its first year the site has been accessed by visitors from every country in the malaria endemic world with the exception of the Central African Republic. High resolution images, in PDF, are necessarily large and can be hard to download so lower resolution PNG versions of every map image are provided for download at low bandwidths.

The full model outputs, posterior predictive distributions, are too large to place online for download (for example, the data for every centile of the posterior distributions of *P. falciparum* endemicity estimates total 100 MB when zipped) and are only useful to groups with expertise in mathematical modelling. Details are advertised on the website [37] and the posterior distributions are sent out on DVD within two weeks of receiving the address of the requestor. Currently, these data are provided at no cost to the requestor and to-date 35 DVDs have been sent out.

### Understanding the data

The data released has been made easy to understand by keeping the structure simple and providing field names in full, avoiding abbreviations and truncations; for example the start date for a particular survey record is covered by the fields “month start” and “year start”. Data are disaggregated to individual sites and dates, and all data are standardized to a common unit so, for example, coordinates are all given in decimal degrees up to five decimal places. Two documents are provided with the Data Explorer; one on how to use the Explorer itself and one about the data. The latter describes the data in more detail, provides a key for the field names and gives information about additional datasets available from other repositories.

Data queries sent to the MAP email address on the website are answered within two weeks. To-date no one has submitted a query about using the survey record data, implying that its structure is intuitive. Queries about the model output data tend to be generic questions relating to i) how to unzip files, ii) how to open/manipulate GIS formatted data and iii) how to open/manipulate the posterior distribution files. There have been no questions about the mapped images to-date.

### Assessing the data

A review of data requests received by MAP in the last twelve months revealed that, in agreement with the consultation group, external groups want to use the data (survey records and modelled data) in their own research which either extends beyond MAP’s own work or explores very different topics, rather than to assess the work MAP has done.

It is inevitable that there are mistakes in the data released and the hope is that the ease of access and comprehension make it easy to assess the data. Each survey record is provided with up to three citations describing the source of the data so users can cross-reference the original source. To-date no group has highlighted mistakes in the survey records but feedback is welcomed and the authors undertake to correct errors that are found and confirmed.

### Sustaining the resource

As time passes, the survey data collated and the modelled data generated will become less relevant to estimates of current malaria risk and control, but more relevant to analyses of the factors that have influenced risk in the past.

The task of sustaining the resource in its current format is made easier by keeping the tools used simple. There is no approval, authentication and authorization system to maintain and the simplicity of the data structure means only a minimal helpdesk function is required.

In the medium term, the MAP group in Oxford, UK is funded to work in this area for the next five years and will add to the database as part of its research. The plans for the longer term include distributing full datasets to a large number of groups: the posterior distributions generated by the models have been sent to four well-established international modelling groups; the survey records, modelled data and mapped data have been provided to the Vector Ecology and Control Network (VECNet) [38]; and the full datasets for Africa have been provided to the African Development Bank-funded Open Data for Africa platform [39] for incorporation into their open database. The process of identifying other repositories for the MAP data will continue as syndication over a broad base will help long-term sustainability.

### Limitation and challenges

The main limitation of this work is the number of data release permissions withheld or pending. A further 12,444 clean and geopositioned parasite prevalence survey records are ready to be released as and when permission from the data contributors is received. It is noticeable that this is only an issue for parasite prevalence data (i.e. infection prevalence in human communities) and not for the vector survey data. Recently collected data may be subject to a release embargo while the data collectors are publishing their own results but historical data have their own issues because it can be difficult to track down the data owner after a number of years have passed. The challenge for the future is to create a culture of routinely sharing data from malaria indicator surveys once the results have been published, as exemplified by MEASURE DHS.

### Comparisons with other online repositories

Most data repositories containing human infectious disease survey data require individual registration and project approval before data can be accessed. One such example is the Global Neglected Tropical Disease Database [40], which currently contains schistosomiasis data that is provided on a survey-by-survey basis. Conversely, the MAP repository releases malaria survey data without the need for prior registration or approval. It also provides a mechanism that allows users to download cross-cutting datasets that incorporate several thousand different surveys.

The prevalence survey records released by MAP overlap with data that can be obtained from the Mapping Malaria Risk in Africa group (MARA) [41], but there are important differences in the two datasets. The primary utility of the MAP repository is as a source of precisely geopositioned survey data linked to a specific point location and date. The MAP team has put considerable effort and resources into disaggregating the survey data available and assigning/checking geopositions. The MARA database is a larger resource of approximately 33,000 parasite prevalence survey records, which includes polygon-located data and aggregate records. It is an Africa-focussed resource with less need to separate parasite species than global resources such as the MAP resource that includes Asia and the Americas and provide species-specific prevalence figures. The MARA database contains data from 39 African countries whereas the MAP database contains parasite data from 80 countries worldwide [see Additional file 2].

The USAID-funded MEASURE DHS repository [30] is an important resource for monitoring vital statistics and public health indicators in low and lower middle income countries [42] that has been available online since 1996 and publicly available via mail since 1984. Huge volumes of individual-level data are freely available from large national household surveys. Many of these surveys include malaria test results (microscopy and/or RDT) and geographical coordinates (randomly displaced by 0 to 2 km for urban locations and 0 to 5 km for rural locations with 1% of rural locations displaced 0 to 10 km), meaning that geolocated survey results are available for over 50,000 survey clusters in 49 countries worldwide. This repository exclusively contains surveys implemented by the MEASURE DHS project so the MAP database complements the MEASURE DHS resource by bringing together other data from a wider range of sources. MEASURE DHS datasets have made a substantial contribution to MAP’s models in the past and will make an even greater contribution in the future (see below).

Vector data can also be obtained from MosquitoMap/VectorMap [43] and VectorBase [44]. VectorMap is an interactive tool that allows users to visualize a wide range of data layers. In the context of occurrence of the dominant malaria vectors, VectorMap does not hold the volume of contemporary occurrence data available from MAP, but VectorMap is broader in scope with a wider range of mosquito genera and species covered, as well as a wider range of diseases. The VectorMap structure contains extra fields that will be useful in the future when new data are available, such as links to GenBank or habitat data.

VectorBase [44] specializes in the collation of genetic data and a new population biology resource is under development that links population genetics to geographical coordinates. VectorMap, VectorBase and MAP have all been developed independently with different but overlapping goals. The hope is that in the future the three resources will form a complementary set that provides vector information linked to geographical coordinates enabling the visualization and analysis of spatial heterogeneity.

### Updating the data

The initial release of parasite prevalence survey data and vector occurrence survey data in October 2012 was based upon the survey data used in five keystone publications [3,17-19,21]. Since this date, new survey records that have not yet been modelled by MAP have been added to the public data release without waiting for MAP to publish its analyses. In the future, new parasite prevalence and vector occurrence data will be added to the online Data Explorer as they come in to MAP or as new permissions to release existing data are received. Much of the confidential dataset currently held was collected by national Ministries of Health. Government departments necessarily have more processes to complete before release permissions can be granted but the hope is that these permissions will be provided in due course and that this resource is as useful for public health agencies as it is for researchers.

The MEASURE DHS project has provided permission for MAP to release site-level (= cluster-level) data that MAP is currently deriving from the individual-level survey data made available by MEASURE DHS. This will result in an increase of a further 3,400 parasite prevalence survey records available from the MAP website in the immediate future, together with identifiers that link these survey data to site information provided by the MEASURE DHS website [30]. The number of MEASURE DHS survey records incorporated will continue to increase over time with the release of new surveys.

Recent research by MAP has produced global spatial estimates of *P. vivax* endemicity [11], sickle cell allele frequency [22] and prevalence of G6PD deficiency [23]. These results will all be made available online in the coming months in the same formats used for the research outputs described above (Table 1). The *P. vivax* survey data will be added to the Data Explorer and the genotype/phenotype survey records that fed into MAP’s human genetic variant research are already available online [45].

In the future, a new collaboration with the WHO aims to support the cartographic analyses underpinning the 2013 World Malaria Report and produce annual malaria burden estimates using extensive data and rigorous modelling techniques (Gething and Cibulskis, personal communication). These estimates will initially be released by the WHO and will then be made available on the MAP website. This work will rely heavily on national malaria surveys and particularly the large volume of data, linked to GPS coordinates, made available by MEASURE DHS [30]. Data sharing with national bodies and international agencies will be essential to ensure the figures produced for and with the WHO are based on the best possible evidence. To this end, all offers of new survey data of any size and format are welcomed.

## Conclusions

It is easy to promise to release data but much harder to deliver. Data for release needs to be clean, the provenance needs to be clear, the structure needs to be readily understood and the data needs to be easy to find for anyone who might want to use it [25]. Collated datasets that bring together data from multiple sources are particularly valuable to public health research and this is particularly true for neglected diseases where there is a paucity of data [40]. Unpublished data that is owned by third parties cannot be released without permission and obtaining permission is a pain-staking and time-consuming task. Research grants rarely include funds for data release, although an expectation of this release may be implicit in the funding terms, and recognition for data release does not match recognition for the publication of results. The release of survey data collated and used by MAP, and of MAP results, was made possible by a dedicated Wellcome Trust Biomedical Resources Grant which addressed the resource issues described above.

Data release, particularly of very large collated datasets, requires effort but this is not a reason to block release. To further biomedical research to improve human health it is imperative that data are shared and MAP’s own research has been made possible by the many hundreds of groups worldwide who originally shared the survey data they had collected. The activities described in this paper mean that a total of 42,090 geopositioned parasite and vector survey results are now available for download from the MAP website plus 3,175 geopositioned survey results for human genetic variants. In addition, 1,634 maps and tables of population estimates derived from MAP’s research are also available as well as the GIS surface data and posterior predictive distributions generated by MAP’s models. There have been calls to support national programmes to map disease risk within their own countries [46] and to build their own online data repositories [47]. The hope is that the work presented here will facilitate both processes; by providing data that national bodies can use to build their own maps and by releasing the source code for the online Data Explorer so others can build similar spatial data repositories. The infrastructure is now in place to deliver survey data and modelled estimates in a timely fashion and MAP aims to double the amount of information available on the website within the next twelve months.

## Abbreviations

MAP: Malaria atlas project; PR: Parasite rate; GIS: Geographical information system; PDF: Portable document format; PNG: Portable network graphics file format; MEASURE DHS: MEASURE Demographic Health Surveys; VECNet: Vector ecology and control network; MARA: Mapping malaria risk in Africa group; WHO: World Health Organization.

## Competing interests

The authors declare that they have no competing interests.

## Authors’ contributions

All the authors worked on the concept for the mechanisms for data release. CLM and WHT designed the online data explorer and WHT developed it and the map production software. AJH adapted the data explorer to accommodate MEASURE DHS data in consultation with CRB and CLM, and developed MAP’s latest research results into maps for online dissemination. CLM wrote the first draft of this manuscript and all authors commented on it. All authors read and approved the final manuscript.

## Supplementary Material

Additional file 1 ***Anopheles *survey data available from the MAP website by species.** The number of data points, defined as a dominant vector species (DVS) occurrence or absence recorded at a unique site-date combination, available online. The full taxonomic descriptions for the 41 DVS, including species, species complexes and subgroups, are given. Click here for file

Additional file 2 ***Plasmodium falciparum *survey data available from the MAP website by endemic country.** The number of survey records (defined as a parasite prevalence survey result for a unique site-date combination) held in MAP’s database and the number of these survey records that are either available online (because they have been published previously or permission has been provided to release them) or are held in confidence. The number of subjects examined for the survey records released is also given. *These data are not visible on the Data Explorer map because the locations are sensitive. Click here for file
